# Supplementary material for: Tissue-selective alternate promoters guide NLRP6 expression
Source: Life Sci Alliance. 2020 Dec 29;4(3):e202000897. doi: 10.26508/lsa.202000897 (PMC7772780; doi:10.26508/lsa.202000897)
Supplement: Supplementary file 2 [file LSA-2020-00897_SdataF2_F3_FS3_FS5.pdf]

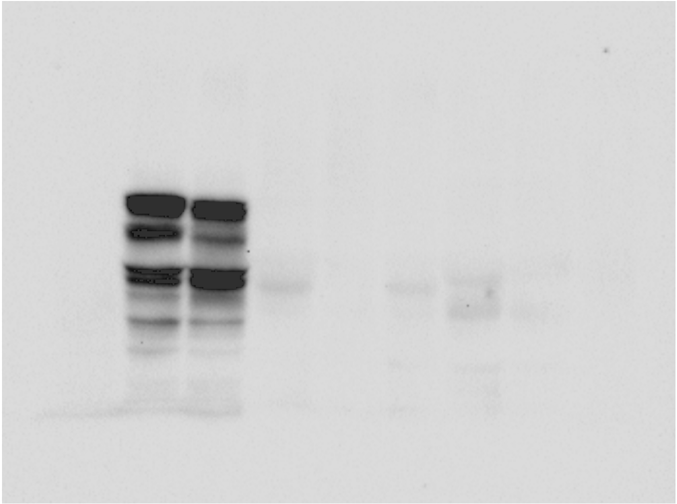

Figure 2

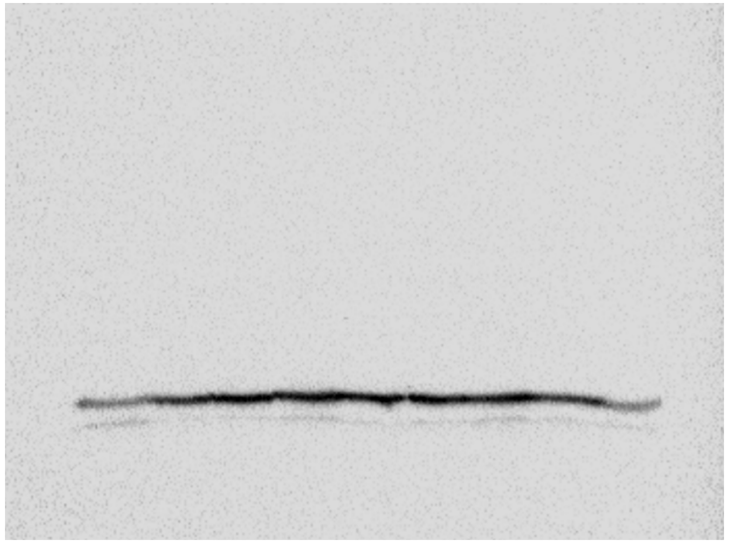

Figure 2

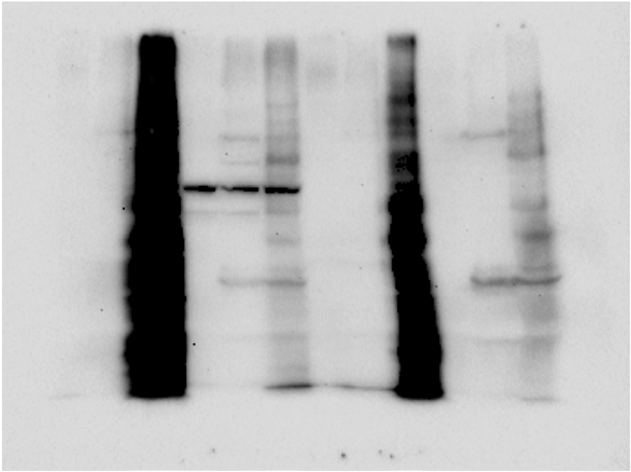

Figure 3

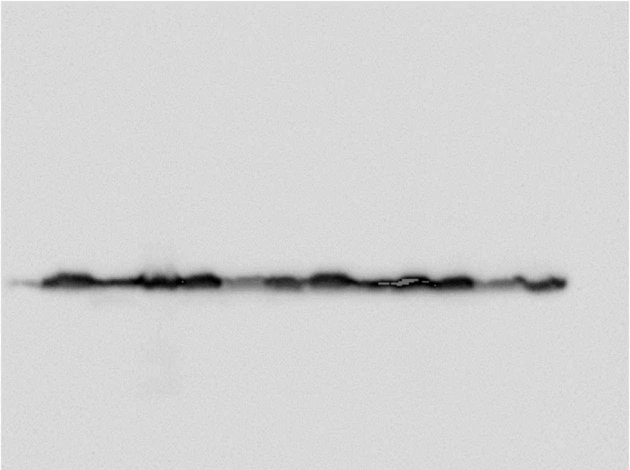

Figure 3

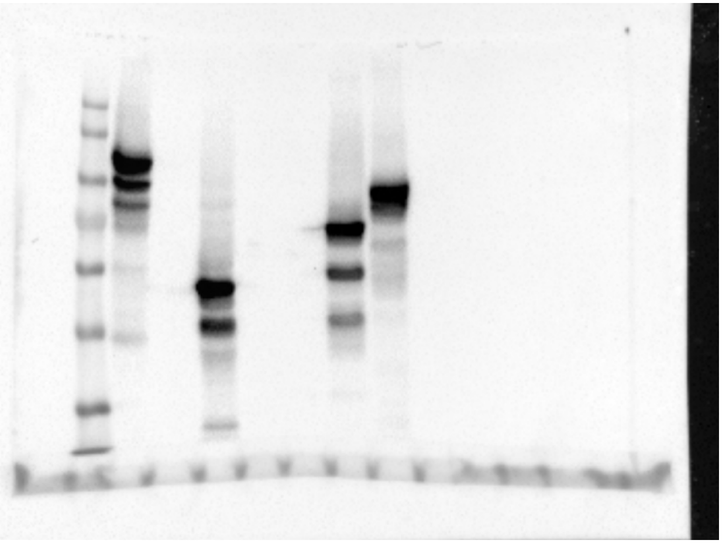

Figure S2a light

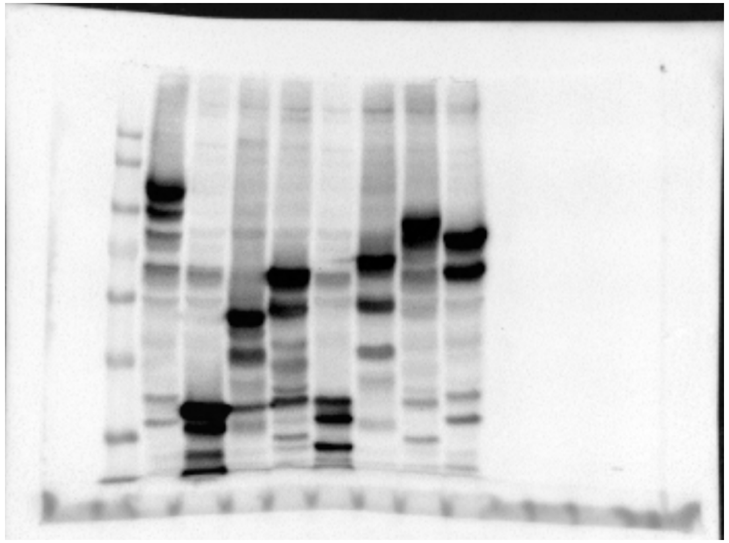

Figure S2a dark

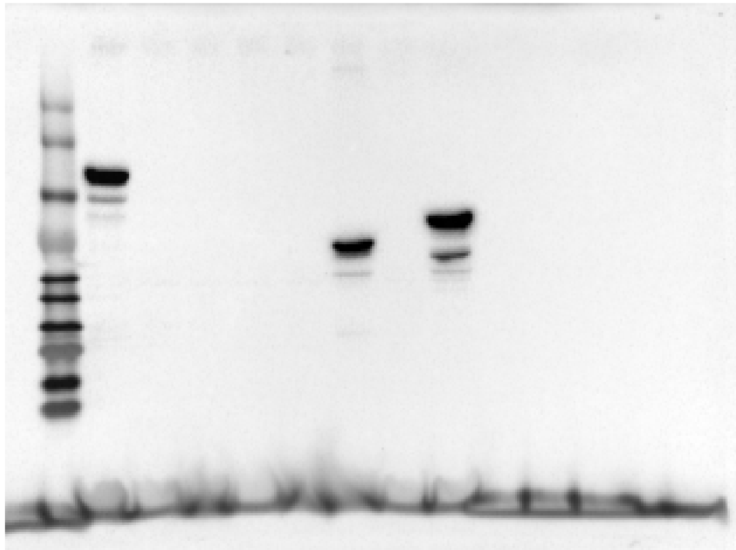

Figure S2b light

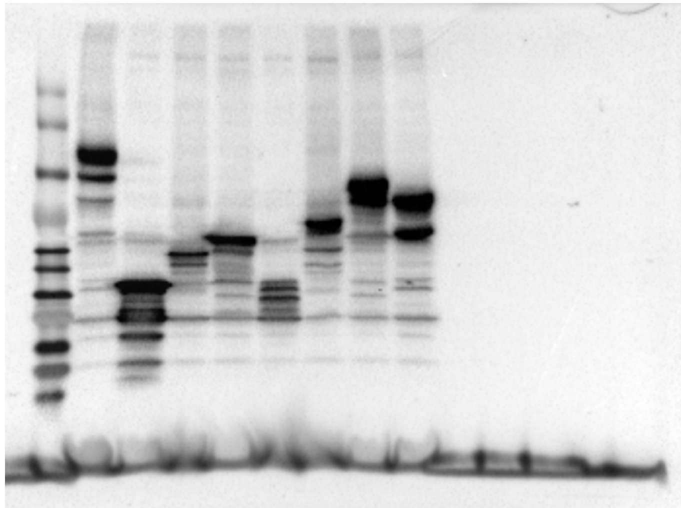

Figure S2b dark

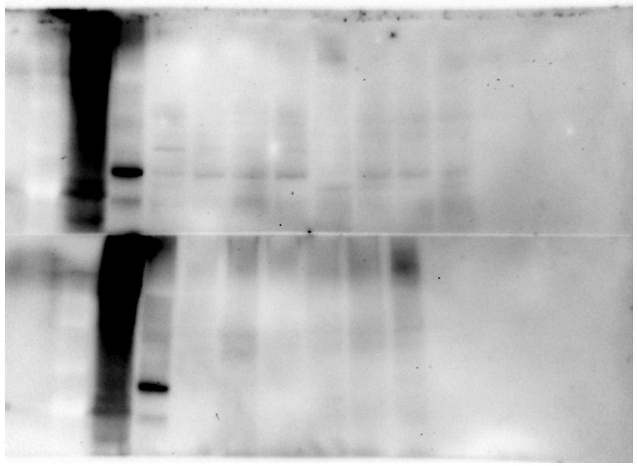

Figure S3 and S5 Nlrp6 dark

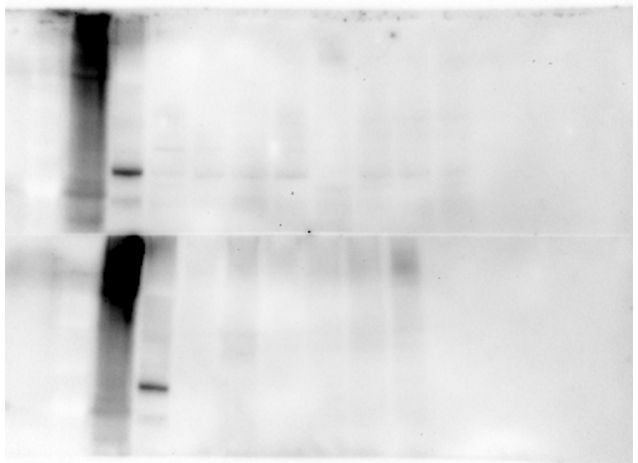

Figure S3 and S5 Nlrp6 light

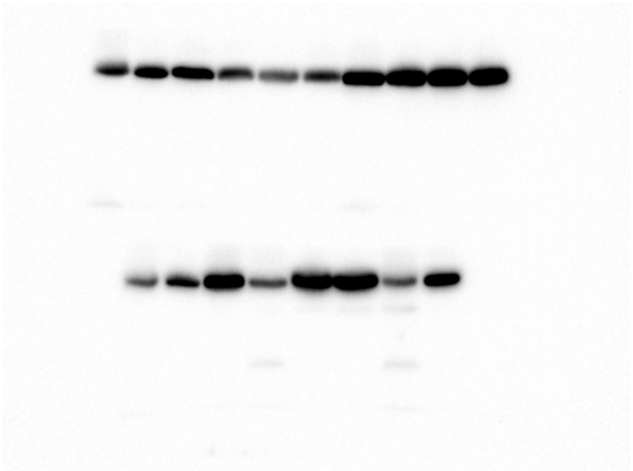

Figure S3 and S5 tubulin

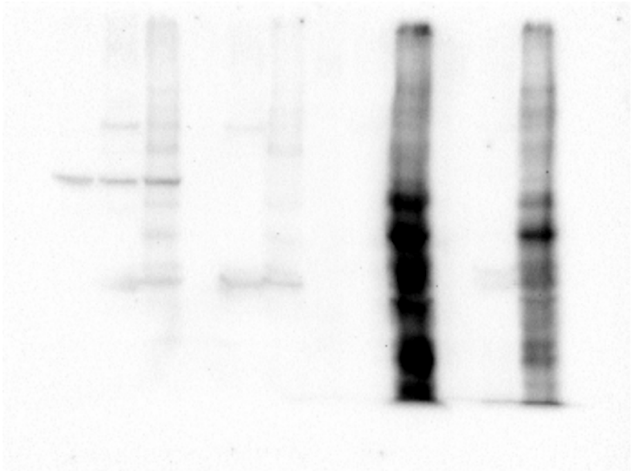

Figure S5 Nlrp6 light

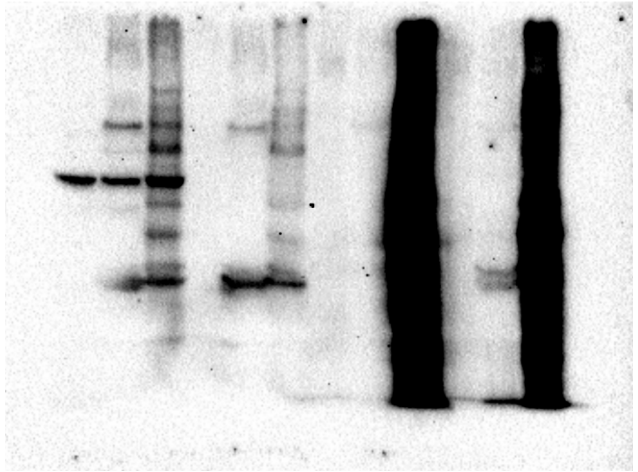

Figure S5 Nlrp6 dark

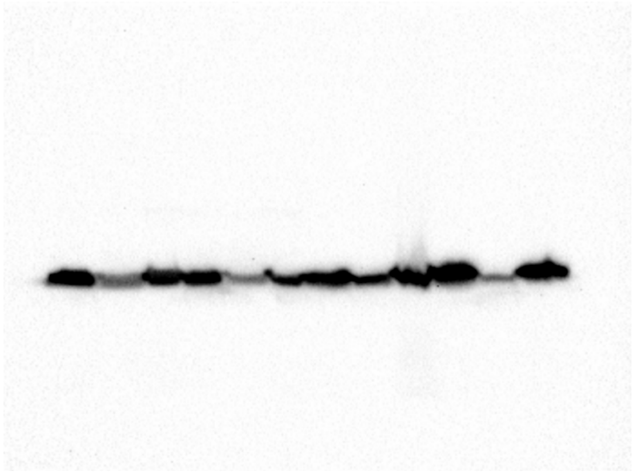

Figure S5 tubulin
